# Cellular Redox Imbalance and Neurochemical Effect in Cognitive-Deficient Old Rats

**DOI:** 10.3390/bs8100093

**Published:** 2018-10-13

**Authors:** Maria Elena González-Fraguela, Lisette Blanco-Lezcano, Caridad Ivette Fernandez-Verdecia, Teresa Serrano Sanchez, Maria de los A. Robinson Agramonte, Lidia Leonor Cardellá Rosales

**Affiliations:** 1 Immunochemical Department, International Center for Neurological Restoration, 25th Ave, Playa, 15805, PC 11300 Havana, Cuba; teresa@neuro.ciren.cu (T.S.S.); robin@neuro.ciren.cu (M.d.l.A.R.A.); 2 Experimental Neurophysiology Department, International Center of Neurological Restoration (CIREN) Ave. 25 No. 15805 e/158 and 160, Playa, Havana 11300, Cuba; lblanco@neuro.ciren.cu (L.B.-L.); ivettef@neuro.ciren.cu (C.I.F.-V.); 3 Physiologic Sciences Department, Latin American Medicine School, Carretera Panamericana, Kilómetro 3 1/2 Municipio Playa, Habana 19148, Cuba; lcardella@infomed.sld.cu

**Keywords:** aging, learning, memory, neurotransmitter, oxidative stress, phospholipase A_2_, serine

## Abstract

The purpose of the present study is to access the linkage between dysregulation of glutamatergic neurotransmission, oxidative metabolism, and serine signaling in age-related cognitive decline. In this work, we evaluated the effect of natural aging in rats on the cognitive abilities for hippocampal-dependent tasks. Oxidative metabolism indicators are glutathione (GSH), malondialdehyde (MDA) concentrations, and cytosolic phospholipase A_2_ (PLA_2_) activity. In addition, neurotransmitter amino acid (*L*-Glutamic acid, γ-aminobutyric acid (GABA), *DL*-Serine and *DL*-Aspartic acid) concentrations were studied in brain areas such as the frontal cortex (FC) and hippocampus (HPC). The spatial long-term memory revealed significant differences among experimental groups: the aged rats showed an increase in escape latency to the platform associated with a reduction of crossings and spent less time on the target quadrant than young rats. Glutathione levels decreased for analyzed brain areas linked with a significant increase in MDA concentrations and PLA_2_ activity in cognitive-deficient old rats. We found glutamate levels only increased in the HPC, whereas a reduced level of serine was found in both regions of interest in cognitive-deficient old rats. We demonstrated that age-related changes in redox metabolism contributed with alterations in synaptic signaling and cognitive impairment.

## 1. Introduction

The demographic changes that affect a large part of humanity are leading to an increase in life expectancy at birth, which has a high impact on societies and their economies. The increase in health costs are not at the expense of the new generation, in fact they are very reduced, but at the expense of the predominance of pathologies related to aging and other physical and intellectual disabilities associated with advanced age.

Aging is an essential factor for many diseases, specifically in neurodegenerative diseases. Oxidative stress constitutes the most important theory of ageing at the molecular level. The cognitive processes that are mediated by the hippocampus (declarative memory) and the prefrontal cortex (working memory) represent the most vulnerable areas to ageing.

This is due to brain cells that are exposed to rising levels of oxidative stress with the loss of energy homeostasis, and the increase in lipid mediators by phospholipases A_2_ (PLA_2_) activity, alterations in neurotransmitters, and signal transduction and accumulation of damage in biomolecules [1].

Oxidative stress is characterized by an imbalance in reactive oxygen species production (ROS) or impaired antioxidant protection. Both mechanisms are considered to have an important role in age-related cognitive decline. Glutathione (GSH) is the main mechanism of antioxidant defense against ROS in biological systems [2]. Glutathione concentration decreases with age in several animal models [3,4,5] and brain areas [6,7]. The reduction in GSH concentration causes an incomplete oxidation of the substrates into the mitochondria inducing the leakage of electrons from the electron transport chain and consequently increasing ROS generation.

A habitual target of ROS are neural membrane lipids, the enhanced phospholipid degradation induces changes in fluidity and permeability in neural membranes with the consequent alteration of ionic channels and modulation in the enzymatic activities coupled to membrane composition [8].

Previous studies have demonstrated the involvement of oxidative signaling pathways in a permanent damage of the neuronal circuitry, especially with glutamatergic neurons. Sustained activation of ionotropic glutamate receptors as the *N*-methyl-d-aspartic acid receptor (NMDAR), could affect intracellular calcium levels leading to activation of proteases, nucleases, and phospholipases [9].

The cytosolic PLA_2_ causes the release of arachidonic acid (AA), hydrolyzing the sn-2 fatty acids of membrane phospholipids. This important polyunsaturated fatty acid (PUFA) is the precursor for the synthesis of eicosanoids and prostanoids. Some findings indicate that PLA_2_ plays important roles in synaptic functions, long-term potentiation (LTP), learning, and memory [10]. The effect of PLA_2_ on cognitive function can be related with the ability of AA to change neuronal metabolism and synaptic activity [11].

Glutamate, an excitatory neurotransmitter in the central nervous system (CNS), plays a recognized role in the regulation of neurogenesis, neuronal survival, synaptic plasticity, and learning and memory processes [12].

Several studies have suggested that the stimulation of PLA_2_ is a process mediated by the glutamate receptor in neuronal cultures [10]. Likewise, D-serine is the most effective co-agonist of the glycine site of NMDAR, which is essential for the optimal function of this receptor in physiological and pathological processes [13].

Experimental data has demonstrated that D-serine treatment decreases the extent of neuron death with a neuroprotective effect against apoptosis [14]. At the present time, serine metabolism is the subject of intense investigation to confirm its role in the diagnosis and therapy of neurological disorders, as well as its therapeutic potential in preclinical models [15,16].

The objective of this study was to obtain evidence about the linkage between dysregulation of glutamatergic neurotransmission in oxidative stress conditions and the role of serine signaling in age-related cognitive decline.

## 2. Materials and Methods

### 2.1. Animal

Male Sprague–Dawley rats obtained from a national breeder institution (CENPALAB, Artemisa) were housed five per cage in a 12:12-h light-dark cycle with food and water ad libitum, under standard conditions for temperature (25 °C) and relative humidity (60%). All experimental studies were performed during light cycle (7.00–19.00 h). We used two age groups: young, (*n* = 30; 2 months old) with a body weight 272 ± 36 g (mean ± standard deviation, SD); and cognitive-deficient old rats (*n* = 45; 23 months old, body weight 520 ± 46 g).

All efforts were made to minimize the number of animals used and their suffering. All procedures concerning animal experimentation were carried out according to the ethical principles for animal research established by the Canadian Council for Animal Care [17] and the Cuban Regulations for the Use of Laboratory Animals (CENPALAB 1997) and were approved by the Ethical Committee of the International Center for Neurological Restoration.

### 2.2. Behavioral Test

Morris Water Maze (MWM)

**Scheme 1:**
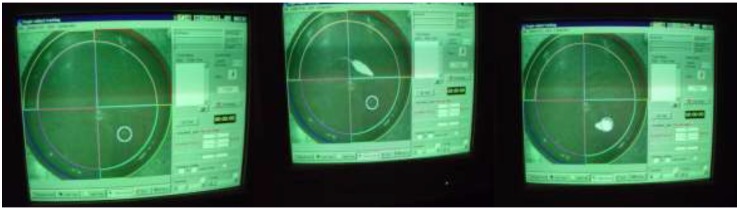
The software display during data acquisition in the Morris water maze (MWM) task using the Spontaneous Motor Activity Recording Tracking (SMART) system from Panlab S.A.

The MWM tests spatial learning and memory retention in rodents and involves an acquisition phase where the animal learns the location of a “goal or escape platform” target over a period of multiple days [18]. An overhead camera and computer assisted tracking system with videomax software (Spontaneous Motor Activity Recording Tracking SMART 2.0) (PanLab, Barcelona España, Spain) was used to record the position of the rat in the maze (Scheme 1). A cued learning test was used to discriminate motoric or motivational factors. On the first day, animals received four trials in a water maze (no distal cues) with a well visible and distinguished platform located at different quadrants.

**Scheme 2:**
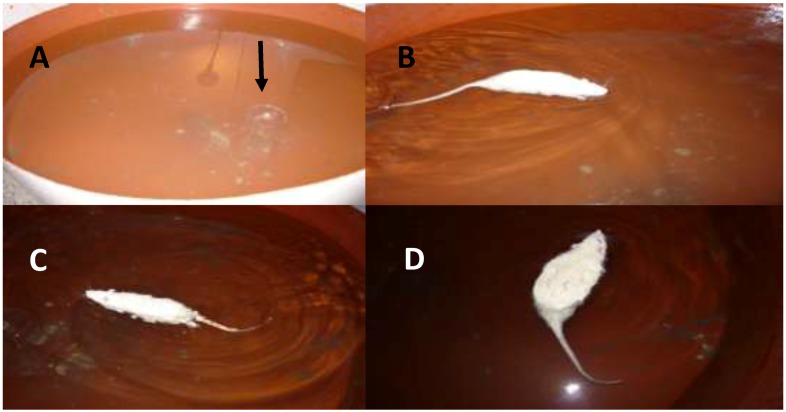
(**A**) The tank before the task. The arrow shows the location of the platform. (**B**–**D**) The different moments in which the rat is searching for the platform in the maze and reaching it.

One day after, a classic spatial acquisition test was introduced. Animals were trained in the MWM with non-visible platform conditions for eight trials per day over four consecutive days. The order of the start position was changed in every trial as well as the sequence of positions along the days. The platform position was kept fixed in the northeast position (NE target quadrant, Q1) throughout the training period (Scheme 2). In each trial, the rat was released from the starting position, facing the wall, and was allowed to search for a hidden escape platform for 60 s. If the animal was unable to find the platform it was gently carried onto it, and allowed a 30 s rest period sitting on the platform. Data from each four trials were grouped into one block (4 days: 32 trials: 8 blocks-B1–B8). On the fifth day (trial 33), the platform was removed and the animal was allowed to search for it for 60 s (probe trial for Q1). This trial evaluated the quality of reference memory. Immediately, the platform was relocated in the same position (Q1) to evaluate four additional trials (34–37, B9) to explore the cognitive flexibility with a previous dissociating stimulus (absence of escape platform). Two days later, the rat’s long-term reference memory was evaluated in a single block of four trials (B10) using the above described paradigm (Q1 at NE position).

Aged rats are classified according to the criteria of 2 or 2.5 standard deviations above the average of the young controls latency [19,20,21].

### 2.3. Sedation and Sample Collection

For the studies that required sedation, an anesthetic mixture of 4 mL of ketalar (ketamine 50 mg/mL, IMEFA, Havana, Cuba), 2 mL of valium (diazepam 5 mg/mL, IMEFA, Havana, Cuba), 1 mL of atropine (atropine, 0.5 mg/mL, IMEFA, Havana, Cuba), and 16 mL of 0.9% sodium chloride solution (0.9% NaCl, IMEFA, Havana, Cuba) was used. The dose regimen was 1 mL of the mixture/100 g of body weight, and immediately the rats were sacrificed by decapitation. Their brains were extracted and rinsed in cold saline; the total frontal cortex (FC) and hippocampus (HPC) were bilaterally dissected. The tissues were frozen in liquid nitrogen, weighed, and stored at −80 °C until the biochemical analysis.

### 2.4. Biochemical Analysis

#### 2.4.1. Oxidative Stress Markers

##### Glutathione GSH Quantification

Tissue samples were homogenized in a glass-Teflon potter containing 5% 5-sulfosalicylic acid (1:15, *w*/*v*) and the protein-free supernatant was isolated by centrifugation at 8160× *g* during 10 min. Total GSH was quantified by Tietze’s recycling assay as described by Azbill et al. [22]. Aliquots of the supernatants were incubated for 25 min at 37 °C in a medium containing 0.21 mM NADPH, 0.6 mM DTNB (5,5’-ditiobis(2-nitrobenzoico), 6.3 mM EDTA (ethylene diamine tetraacetic acid), and 143 mM sodium phosphate pH 7.5. Following the addition of 0.5 U GRD (glutathione reductasa), the absorbance increase at 412 nm due to the formation of 5-thio-nitrobenzoate was recorded. The concentration values were calculated from a GSH standard curve.

##### Malondialdehyde (MDA) Quantification

Tissues samples were homogenized in 1 M Tris/0.25 M sucrose buffer (pH 7.4) at a tissue/buffer volume ratio of 1/5. The homogenates were centrifuged at 14,000 rpm for 15 min. Two-hundred microliters of the sample were vortexed with 400 μL of a 0.67% thiobarbituric acid solution in 0.2 M HCl, then incubated for 15 min in a water bath at 100 °C and centrifuged at 2448× *g* for 10 min at room temperature. The resulting supernatant was used for measuring absorbance at 535 nm. The concentration values were calculated from an MDA standard curve [23].

##### Assay for Calcium-Dependent Cytosolic Phospholipase A2 (PLA2) Activity

PLA_2_ enzymatic activity was measured using the protocol recommended by the manufacturer (Cayman Chemical Company, Ann Arbor, MI, USA). Briefly, 10 μL sample were incubated with 1.5 mM arachidonoyl thio-phosphotidylcholine (a PLA_2_ substrate) for 1 h at room temperature. Enzyme catalysis was stopped by the addition of beta dystrobrevin/EGTA (Ethylene glycol-bis(2-aminoethylether)-*N*,*N*,*N*′,*N*′-tetraacetic acid), and the optical density of the sample was measured at 405 nm. PLA_2_ activity was calculated in accordance to kit instruction and PLA_2_ activity was expressed in U/mg [24].

#### 2.4.2. Amino Acid Analysis

##### Sample Preparation and Derivatization

The brain tissue for amino acid analysis was homogenized in ice-cold 0.2 M perchloric acid including cysteine (4 mL per g of tissue) and sonicated for 5 min. The brain homogenate was centrifuged at 17,000 rpm for 4 min at 4 °C and the supernatant was diluted (1/50), aliquoted, and stored at −20 °C. 20 µL of standard (*L*-Glutamic acid, γ-aminobutyric acid (GABA), *DL*-Serine and *DL*-Aspartate) or sample was added with 20 μL of the derivatization reagent (25 mg OPA (Ophthaldialdehyde) of diluted in 1500 μL of methanol, 100 μL of borate buffer 0.1 M (pH9), and 30 μL of β-mercaptoethanol), the derivatization mixture was vortexed for 1 min at room temperature, After that, 5 μL of 5% acetic acid were added to stop of reaction, then, 20 μL of this final solution was injected into the HPLC system.

##### Instrumentation and Chromatographic Conditions

For chromatographic analysis, an HPLC system (Agilent 1200 Technologies) (Santa Clara, CA, USA) was used. Chromatographic separation was achieved on a reversed phase column (HR-80, ESA), using an isocratic chromatographic pump (Agilent 1200 series), and detected with a fluorescence detector (Agilent G1321A, 1100 series) set at λ_excitation_ 340 nm and λ_emission_ 460 nm. The column temperature was maintained at 25 °C and the mobile phase was pumped at a flow rate of 1.0 mLmin^−1^. The chromatographic data were recorded using the Agilent ChemStation B.04.02SP1 software. The mobile phase was composed of 0.1 M NaH_2_PO_4_/25% methanol.

### 2.5. Data Processing and Statistical Analysis

The values were expressed as mean ± standard error mean. Normal distribution and homogeneity of variance of the data were tested by the Kolmogorov–Smirnov and Levene tests, respectively. The comparison between experimental groups of the biochemical indicators was performed by a one-way analysis of variance (ANOVA) and the behavioral variables in the MWM were analyzed by analysis of variance for repeated measurements (ANOVA). For post hoc comparisons was used the Tukey honest significant differences test (HSD) and nonparametric comparison with Mann–Whitney U test. Significant differences were considered only if *p* < 0.05.

## 3. Results

### 3.1. Behavioural Studies

#### Morris Water Maze

**Figure 1:**
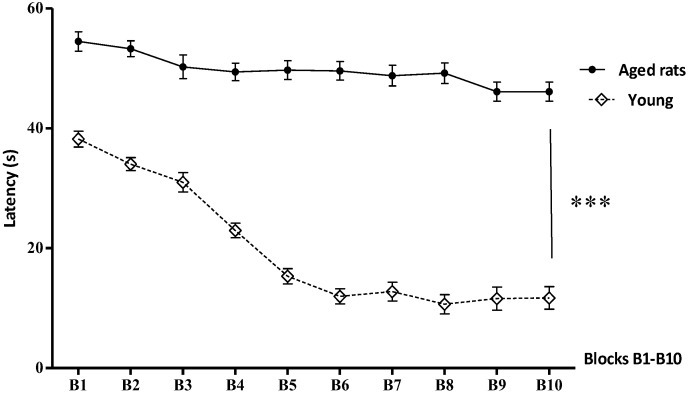
Latency in the MWM (mean ± SD mean are represented) to find the hidden platform during the training sessions grouped into the blocks (B). Classical training (B1–B9) and long-term contextual memory (B10). *** Significant differences between experimental groups: aged rats (*n* = 45) and young (*n* = 30) (*p* < 0.001).

**Figure 2:**
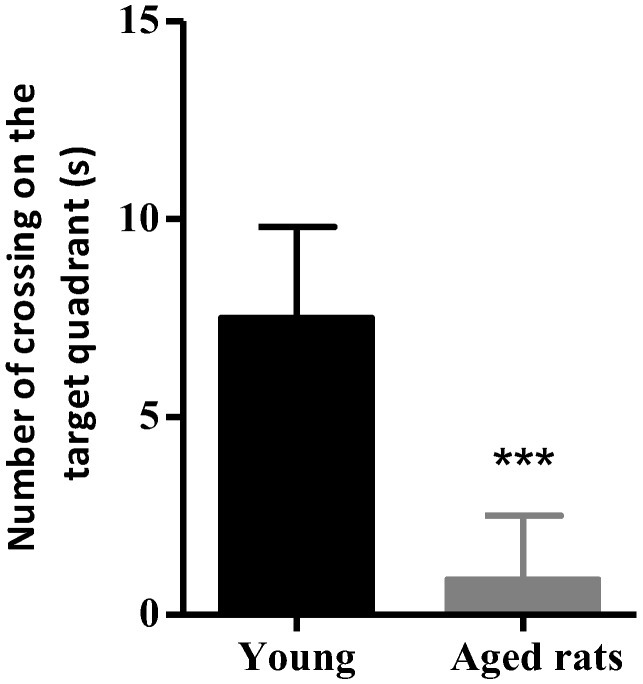
Number of crossings on the target quadrant (mean ± SD mean are represented) *** Significant differences between experimental groups: aged rats (*n* = 45) and young (*n* = 30) (*p* < 0.001).

The post-hoc analysis on spatial long-term memory revealed significant differences between experimental groups for escape latency to platform (F_(1, 28)_ = 12.39; *p* < 0.001) related with an increase in aged rats that demonstrated severe spatial memory impairment in this group (Figure 1). Likewise, regarding the number of crossings, the aged rats exhibited a poor performance in the acquisition of the navigation search strategy during the test. The Tukey honest significance differences test, showed a reduction of crossings and time spent on the target quadrant in older animals than younger rats (F_(1, 28)_ = 8.91; *p* < 0.001) (Figure 2, Table 1).

### 3.2. Biochemical Indicators

The comparison between experimental groups with regard to GSH content showed significant differences in examined brain areas in cognitive-deficient old rats (FC (Z = 5.41; *p* < 0.001); HPC (F_(1,38)_ = 288.6; *p* < 0.001)). The level of GSH dropped by 82% in FC and 80% in HPC in respect to the control groups. These results are associated with a significant increase in MDA concentrations for aged rats only for hippocampus, a highly sensitive region to oxidative stress. (HPC (F_(1,38)_ = 837.9; *p* < 0.001)).

**Figure 3:**
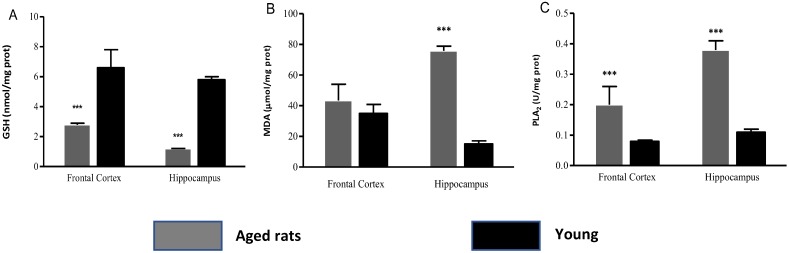
Effect of aging on oxidative metabolism indicators in the examined brain areas. (**A**) Glutathione (GSH), (**B**) Malondialdehyde (MDA), (**C**) Phospholipase A2 (PLA2). *** Corresponds to significant differences between experimental groups (*p* < 0.001).

Similarly, a considerable increase was observed in PLA_2_ activity for analyzed brain areas. The enzyme activity revealed significant differences among experimental groups (FC (F_(1,38)_ = 29.15; *p* < 0.001); HPC (F_(1,38)_ = 70.96, *p* < 0.001)) in correspondence with the reduction of GSH content in the studied brain structures GSH: Glutathione, MDA: Malondialdehyde, PLA_2_: Phospholipase A_2_, GABA: γ-aminobutyric acid. (Figure 3, Table 2).

#### Amino Acid Concentrations

**Figure 4:**
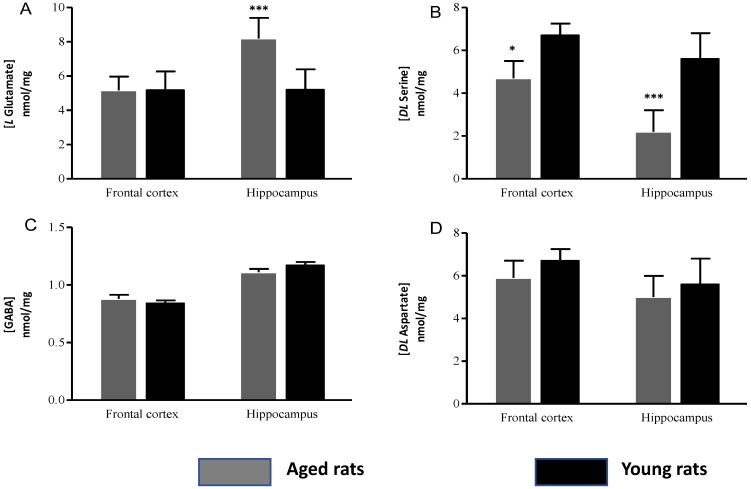
Effect of aging on amino acid concentration in the examined brain areas. (**A**) *L* Glutamate, (**B**) *DL* Serine, (**C**) GABA, (**D**) *DL* Aspartate. *** (*p* < 0.001); * (*p* < 0.05) corresponds to significant differences between experimental groups.

The levels of GABA were unchanged compared with controls in brain regions investigated in aged rats (FC (F_(1,38)_ = 0.17; *p* > 0.05) and HPC (F_(1,38)_ = 0.39; *p* > 0.05)). Furthermore, the aspartate concentrations were not significantly different between groups (FC (F_(1,38)_ = 0.18; *p* > 0.05) and HPC (F_(1,38)_ = 0.70; *p* > 0.05)). In contrast, the levels of glutamate only increased in HPC in aged rats (HPC (F_(1,38)_ = 93.32; *p* < 0.001), whereas a reduced level of serine was found in the regions of interest in cognitive-deficient old rats (FC (F_(1,38)_ = 58.3; *p* < 0.001) and HPC (F_(1,38)_ = 365.1; *p* < 0.001)) (Figure 4, Table 2).

## 4. Discussion

Some aged animals undergo severe cognitive deficiencies, whereas others preserve their normal cognitive abilities. In particular, spatial memory appears to be significantly affected [25]. The behavioral results allowed us to select aged rats that demonstrated a deficient execution in the acquisition of a successful navigation strategy in the MWM. The metabolic changes originated by GSH depletion in HPC caused increased swim times in the MWM test associated with a reduction of time spent on the target quadrant, confirming a failure in the memory trace formation, and therefore cognitive dysfunction.

We observed a significant GSH depletion in cognitive-deficient old rats, as much in FC as in HPC. Besides its roles in maintenance of oxidant homeostasis within the cell, GSH may serve as a substrate and an allosteric modulator of eicosanoid biosynthesis, in the regulation of NMDAR activity and activation of transcription factors [26]. We found a reduction of more than 70% in the GSH content in cognitive-deficient old rats compared to the young rats. The majority of authors have reported a decreases of tripeptide in brain tissue of around 50% in previous rodent studies [27,28].

The strong GSH decrease observed in our work generates hydrogen peroxide (H_2_O_2_) and hydroxyl radical (OH) that induce lipid peroxidation in the membrane of neurons and glial cells and damage the (Ca^2+^ and Na^+^/K^+^) ATPases and glucose transporters [29]. In connection with this result, we observed a significant increase in MDA concentration and PLA_2_ activity in aged rats for examined brain areas. The loss of the energy metabolism homeostasis, redox imbalance, and subsequent damage by accumulation of MDA could originate the functional impairment in aged rats.

Due to its numerous functions for maintenance of membrane phospholipid balance and for originating several lipid mediators, PLA_2_ activation has been implicated in pathological conditions in the CNS for neurodegenerative diseases among others [8,30,31,32].

H_2_O_2_ accumulation induces the redox ambience appropriate for activating the PLA_2_ pathway by means of extracellular signal-regulated kinase (ERK) and interleukin 1b [33,34]. In this context, PLA_2_ activity increases the release of AA from the plasma membrane, which directly produce 4-hydroxynonenal and MDA.

Arachidonic acid AA undergoes oxidative modifications and increases the mitochondrial ROS levels in the hippocampus and cerebral cortex [35]. As such, the AA and its metabolites are permeable to the membrane and could affect the function of neighboring neurons with disruption of monolayer integrity and cell death [36].

The results obtained from the cognitive-deficient old rats revealed high PLA_2_ enzymatic activity and consequently increased AA generation. It is important to point out that high concentrations of AA cause uncoupling oxidative phosphorylation with a detrimental effect on the ATP-producing capacity of mitochondria, which results in mitochondrial dysfunction and an additional increase in the production of ROS.

These experimental data are in line with clinical studies. Jiang R et al. [37] reported that higher PLA_2_ enzymatic activity is associated with increased prevalence of cognitive impairment in the Chinese population. On the other hand, there is evidence that the contribution of PLA_2_ activity in regulating neuronal excitatory functions, in cultured primary cortical neurons, trigger NMDAR receptors, which has been demonstrated to activate PLA_2_ and AA release [38]. In turn, PLA_2_ activity and AA are involved in receptor signaling which is linked with oxidative events which underline important roles in synaptic signaling, LTP, learning and memory [39,40].

In consonance with these studies, the present findings evidence an increase in glutamate concentrations in the HPC of aged rats which is closely associated with the elevation of PLA_2_ activity in this region. The LTP dependence of NMDAR activation has been demonstrated in several brain areas, including hippocampal subfields like the dentate gyrus and the CA1 [41].

It is well established now that excessive levels of glutamate can produce toxic effects due to overstimulation of NMDAR, increasing the intracellular Ca^2+^ and activate Ca^2+^-dependent enzymes including PLA_2_. This cascade of events culminates with a Ca^2+^ dysregulation in neurons and astrocytes, which initiates cellular death by a process known as excitotoxicity. This process implicates the activation of proteases, the generation of radical species, and mitochondrial Ca^2+^ overload that affects critical biological molecules including lipids, proteins, and DNA.

However, on the other hand, one of the deleterious consequences of GSH metabolism dysfunction is the NMDAR hypoactivity [42]. The GSH insufficiency in aged rats could induce the formation of disulfide bonds between pairs of cysteine residues on the extracellular portion of the NMDAR generating a selective reduction of NMDAR currents [42], which contrast with the possible excitotoxic effect of NMDAR activation in old rats due to high levels of glutamate.

Previous studies with NMDAR blockade suggested that altered redox states of these extracellular cysteine residues could mediate the decline in NMDAR function during aging [43]. The application of dithiothreitol, a specific reducing agent, increased the NMDAR component and the magnitude of LTP in hippocampal slices from old rodents [43].

The intracellular redox status observed in the aged rats can directly contribute to synaptic inefficiency of NMDAR, caused by changes in neurotransmitter systems in vulnerable brain regions. Liu et al. [44] have reported that H_2_O_2_ accumulation is associated with the downregulation of the GluN2 subunits of the NMDAR in adult rat hippocampi. Otherwise, recent modeling work has shown that reduced NMDAR signaling into interneurons produced alterations in spatial representations that affected working memory execution [45]. Therefore, the suppression of NMDAR responses could induce changes in memory mechanisms that impaired the cognitive processes in GSH-depleted aged rats.

This previous hypothesis is supported by a reduced level of serine in the brain regions investigated in aged rats. D-serine, an enantiomer of serine, has a crucial characteristic of binding to NMDAR [46]. This amino acid binds to the glycine-site of the GluN1 subunit of NMDAR so that glutamate can link to GluN2 subunits [47].

We found a reduction of around 60% in serine levels in HPC and 30% in FC in cognitive-deficient old rats with regard to younger counterparts. The bond of D-serine is critical to synaptic NMDAR function [48] due to D-serine having three additional hydrogen bonds to the receptor, which may activate the NMDAR more efficiently than glycine [49]. This finding increases the possibility that alterations in serine metabolism could be a contributor to the dysfunctions of memory in the aged rats.

D-serine is converted from L-serine by serine racemase, an enzyme dependent of pyridoxal phosphate, mostly expressed in excitatory neurons in the CNS of rodents [50]. D-serine is especially increased in brain areas involved in cognitive processes, as in the cerebral cortex, hippocampus, and basal ganglia [49].

The pathway responsible for the degradation of D-serine in the CNS is the D-amino acid oxidase (DAAO) [51]. Nevertheless, the reduction in serine concentration could involve an exceptional property of serine racemase which is able to catalyze the degradation of D-serine and L-serine to pyruvate and ammonia [13].

The possibility of serine racemase to enhance pyruvate levels from metabolic availability of serine may represent an important mechanism to obtain energy for mitochondrial oxidative phosphorylation and synthesis of ATP. This alternative of the serine metabolic pathway explains how the increased ROS production secondary to GSH depletion observed in aged rats reduces the synaptic levels of D-serine and exacerbates the NMDAR dysfunction in increased energy demand conditions.

These results are coincidental with reports from others authors who have shown that alterations of LTP expression are associated with a deficiency of NMDAR [52,53,54] and is recovered in aged rats and senescence-accelerated mice by D-serine treatment [54].

## 5. Conclusions

These findings suggest that age-related changes in the cellular redox metabolism contributes to memory dysfunctions and cognitive decline. The energy demands of synaptic activity lead to increased ROS production as a by-product of ATP synthesis with elevated utilization of a glutathione-based antioxidant system. Glutathione depletion induces an acute increase in the oxidative stress conditions and may generate NMDAR hypofunction and alterations in synaptic transmission. In the same way, the reduction of serine levels in aged rats, as the most effective co-agonist, could lead to a marked impairment of NMDAR function. The results discussed here support the hypothesis of redox homeostasis as a crucial element that regulates brain function and it will be necessary to further research the molecular mechanism of observed results.

## Figures and Tables

**Table 1:** Data (mean ± SD mean) from four trials grouped in blocks (B1–B10) for the *latency* in the MWM and the number of crossings in the target quadrant between experimental groups.

Blocks (B)	Aged Rats (Mean ± SD Mean)	Young (Mean ± SD Mean)
B1	54.5 ± 1.62	38.20 ± 1.32
B2	53.30 ± 1.33	34.02 ± 1.087
B3	50.26 ± 1.97	30.99 ± 1.61
B4	49.41 ± 1.48	22.96 ±1.20
B5	49.7 ± 1.56	15.30 ± 1.27
B6	49..59 ± 1.54	11.96 ± 1.26
B7	48.8 ± 1.70	12.75 ± 1.57
B8	48.2 ± 1.73	10.66 ± 1.59
B9	46.12 ± 1.60	11.58 ± 1.94
B10	46.06 ± 1.7	11.70 ± 1.92
Number of Crossings	0.9 ± 1.6	7.5 ± 2.3

**Table 2:** Data (mean ± SD mean) corresponding to biochemical indicators in examined brain areas between experimental groups.

Biochemical Indicators	Frontal Cortex Aged Rats	Frontal Cortex Young Rats	Hippocampus Aged Rats	Hippocampus Young Rats
GSH	1.3 ± 0.1	5.45 ± 1.2	0.76 ± 0.22	5.15 ± 0.2
MDA	43.42 ± 10.6	34.93 ± 5.95	80.31 ± 3.01	15.84 ± 2.02
PLA_2_	0.2 ± 0.06	0.08 ± 0.0	0.35 ± 0.03	0.11 ± 0.01
L Glutamate	5.29 ± 0.8	5.17 ± 1.10	10.24 ± 1.2	6.89 ± 1.2
GABA	0.88 ± 0.03	0.94 ± 0.02	1.11 ± 0.03	1.17 ±0.03
DL Serine	4.85 ± 0.8	6.07 ± 0.55	2.13 ± 1.02	5.52 ± 1.2
DL Aspartate	5.35 ± 0.72	5.4 ± 0.55	5.02 ± 1.03	5.16 ± 1.2
